# A Possible Regression Equation for Predicting Visual Outcomes after Surgical Repair of Open Globe Injuries

**DOI:** 10.1155/2017/1320457

**Published:** 2017-01-12

**Authors:** Huseyin Gursoy, Mustafa Deger Bilgec, Afsun Sahin, Ertugrul Colak

**Affiliations:** ^1^Department of Ophthalmology, Eskisehir Osmangazi University Medical Faculty, Eskisehir, Turkey; ^2^Department of Biostatistics, Eskisehir Osmangazi University Medical Faculty, Eskisehir, Turkey

## Abstract

*Background*. To analyze the effects of factors other than the ocular trauma score parameters on visual outcomes in open globe injuries.* Methods*. Open globe injuries primarily repaired in our hospital were reviewed. The number of surgeries, performance of pars plana vitrectomy (PPV), lens status, affected tissues (corneal, scleral, or corneoscleral), intravitreal hemorrhage, intraocular foreign body, glaucoma, anterior segment inflammation, loss of iris tissue, cutting of any prolapsed vitreous in the primary surgery, penetrating injury, and the time interval between the trauma and repair were the thirteen variables evaluated using linear regression analysis.* Results*. In total, 131 eyes with a mean follow-up of 16.1 ± 4.7 (12–36) months and a mean age of 33.8 ± 22.2 (4–88) years were included. The regression coefficients were 0.502, 0.960, 0.831, −0.385, and −0.506 for the performance of PPV, aphakia after the initial trauma, loss of iris tissue, penetrating injury, and cutting of any prolapsed vitreous in the primary surgery, respectively (*P* < 0.05 for these variables).* Conclusions*. The performance of PPV, aphakia after the initial trauma, and loss of iris tissue were associated with poor visual outcomes, whereas cutting any prolapsed vitreous in the primary repair and penetrating-type injury were associated with better visual outcomes.

## 1. Introduction

Open globe injuries are some of the most common causes of irreversible vision impairment worldwide [1]. All the structures of the eye, including the anterior segment, the posterior segment, and the extraocular structures, can be damaged following ocular trauma [2, 3]. Thus, all the ophthalmology subspecialties, including the departments of vitreoretinal diseases, corneal diseases, and cataracts, can be involved in the proper management of serious ocular injuries [2–4]. Analysis of serious ocular trauma is difficult because any of the internal and external eye structures can be critically damaged; the large extent of possible injuries ensures that similar groups of eye injury cases are required for comparative studies analyzing the prognostic factors and management strategies in open globe injuries [2–5]. In addition to these factors, it is difficult to discuss the clinical relevance of visual predictors in ocular trauma reports because most of the factors affecting the visual prognosis overlap.

Kuhn et al. evaluated greater than 2,500 injuries and defined an ocular trauma score (OTS) [6]. The OTS is calculated using six parameters, including the initial visual acuity, occurrence of globe rupture, endophthalmitis, perforating injury, retinal detachment, and afferent pupillary defects [6]. Several variables other than the OTS parameters have been studied and serve as additional significant predictors of the visual outcomes in globe injuries [7–11].

The aim of our study was to analyze the effects of factors other than the OTS parameters on final visual outcomes in open globe injuries.

## 2. Materials and Methods

In this study, we reviewed the medical records of patients at the Eskisehir Osmangazi University Hospital of Faculty of Medicine to whom emergent eye surgery was suggested for initial treatment from January 2012 to April 2015. The study was approved by the Institutional Review Board (2016-25). The data collection conformed to all the local laws and was compliant with the principles of the Declaration of Helsinki. The inclusion criteria for this study were as follows: (1) the patient was capable of describing his/her vision at the initial and final examinations; (2) a primary repair of an open globe injury was performed at our hospital; (3) complete charts were accessible, including the OTS parameters and details of the surgeries performed; and (4) follow-up of at least 12 months occurred.

Informed consent was obtained from all the subjects or their parents before we performed each treatment. All primary surgeries were performed by experienced ophthalmologists (H.G., M.B., and A.S.) under general anesthesia. The other surgeries, specifically pars plana vitrectomy (PPV) procedures, cataract surgeries, and evisceration of the globe, were performed by the vitreoretinal, cataract, and oculoplastic services of our department, respectively.

Computed tomography of the head and orbit was performed for all patients over 17 years of age to exclude foreign bodies and orbital-wall fractures. Postoperative care was applied based on previous studies [12, 13]. Systemic prophylactic ciprofloxacin (750 mg q 12 hr) was administered to all patients over 18 years of age and used for one week. Amoxicillin and clavulanate potassium were preferred in patients younger than 18 years of age. Topical steroids (dexamethasone 0.1% eye drop q 4 hr) and fortified topical antibiotics (vancomycin 25 mg/ml q 1 hr and ceftazidime 50 mg/ml q 1 hr) with mydriatics (Cyclopentolate 1% eye drops q 12 hr) were applied in all cases for 10 days. All the drops were tapered off over the next three weeks [12, 13]. The corneal sutures were removed three to six months after the primary repair if additional surgeries were not undertaken. The corneal sutures were left for a minimum of three months because the fibrosis of the corneal wound occurs at least three months after the primary repair [12]. The OTS parameters (initial visual acuity, rupture of the globe, endophthalmitis, perforating injury, retinal detachment, and relative afferent pupillary defect) were noted in all cases. Best-corrected visual acuity (BCVA) was tested using the Snellen eye chart at 6 m. The initial and final BCVAs in logMAR units were noted in all cases. For the data analysis in the study, 2.7 logMAR, 2.8 logMAR, and 2.9 logMAR were used instead of hand movement (HM), perception of light (PL), and no perception of light (NPL), respectively [13, 14]. The intraocular pressure (IOP) was measured using the Tono-Pen at each visit following the primary surgery. Glaucoma was defined as a raised IOP (>21 mmHg) and the requirement of antiglaucomatous drugs one month or more after the primary repair. The age, number of surgeries, PPV performance, lens status, affected tissues (corneal, scleral, or corneoscleral), intravitreal hemorrhage (IVH), an intraocular foreign body, glaucoma, anterior segment inflammation, the loss of iris tissue at the initial trauma, the cutting of any prolapsed vitreous in the primary surgery, a penetrating injury, and the time interval between the trauma and repair represent the thirteen independent variables evaluated in the study. Evisceration of the globe was not included in the number of surgeries performed for each case. The loss of iris tissue was defined as a prolapsed iris at the time of injury or a necrotic iris cut during the primary repair. The following lens statuses were noted: normal, traumatic cataract, and aphakia after the initial trauma. Aphakia after the initial trauma was defined as the lens being fully prolapsed out of the globe (missing or under the conjunctiva) at the time of injury or the removal of the disrupted lens during the primary repair. Surgery was performed in cases of a traumatic cataract if cataract surgery was considered beneficial to the visual outcome. Anterior segment inflammation was defined as noninfectious inflammation detected biomicroscopically in the anterior segment structures one month or more after the primary repair.

### 2.1. Statistical Analyses

The paired sample *t*-test was used to compare the initial BCVAs with the final visual outcomes. The final visual outcomes were compared among cases over 17 years old, between 7 and 17 years old, and under 7 years old using one-way ANOVA. The relationship between the OTS and the visual outcomes was determined using univariate linear regression analysis. A multiple linear regression analysis was performed to demonstrate the effects of the thirteen independent variables on the final visual outcome. The multicollinearity among the independent variables was measured using the VIF (variance inflation factor) scores. All the VIF scores were <5, which indicated that these variables were uncorrelated. A model was constructed using the final visual outcome as a dependent variable and our thirteen factors as independent variables. The backward stepwise elimination method was used to identify the influences of potential confounders and their statistical significance on the visual outcome, and this was followed by the formation of a regression equation. The influence of the significant variables according to the multiple linear regression model was analyzed with an independent sample *t*-test. Statistical analyses were performed using IBM SPSS Statistics version 21.0. *P* values < 0.05 were required for statistical significance.

## 3. Results

The record review identified one hundred fifty-two patients. One hundred thirty-one open globe injuries (sixty-three right and sixty-eight left eyes) satisfied the inclusion criteria. Ninety-six patients (73%) were male. Ninety-nine (75.6%) patients were over 17 years old, nineteen patients (14.5%) were between 7 and 17 years old, and thirteen patients (9.9%) were under 7 years old. The mean final BCVA in logMAR units was 1.53 ± 1.13, 1.14 ± 1.12, and 1.11 ± 0.65 in cases >17 years old, between 7 and 17 years old, and <7 years old, respectively (*P* = 0.201). The mean follow-up time was 16.1 ± 4.7 (12–36) months. Patching was prescribed in eight patients under 9 years old. The OTS for each case was calculated according to the findings in Table 1.

The mean OTS was 53.8 ± 14.3 (26–90). The effect of the OTS on the visual outcome was significant based on univariate linear regression, and the coefficient of variation was 0.281 (*P* < 0.001). The regression coefficient for the OTS was −0.039, which indicated that a one-unit increase in the OTS resulted in a 0.039-unit decrease in the visual acuity in logMAR.

The mean initial BCVA in logMAR units was 2.47 ± 0.61 (0.15–2.90), whereas the mean final BCVA was 1.43 ± 1.10 (0–2.90) (*P* < 0.001). The final BCVA was NLP in fourteen (10.7%), LP in seven (5.3%), HM in twenty-five (19.1%), 19/200–1/100 Snellen in twenty-two (16.8%), 4/10–1/10 Snellen in thirty-two (24.4%), and ≥5/10 Snellen in thirty-one (23.7%) eyes. Evisceration was performed in six cases.

The independent variables evaluated with a multiple linear regression analysis are presented in Table 2.

The cutting of any prolapsed vitreous in the primary surgery was performed in all eyes in which this pathology existed or where it was possible to visualize. The procedures performed other than primary repair included cataract surgeries, PPVs with or without a lensectomy, and anterior segment revision procedures. Sixteen of the PPV procedures and twenty-three of the cataract surgeries were performed under local anesthesia. A PPV was performed in thirty-two eyes. In six eyes, a revision PPV was required. The indications for PPV were retinal detachment, IVH, and an intraocular foreign body in twenty-one, thirteen, and seven of the eyes, respectively. Retinal detachment and IVH were combined in five cases. An intraocular foreign body was associated with retinal detachment and IVH in three eyes and one eye, respectively. The time interval between the trauma and repair was greater than 13 hours in 23 eyes (17.6%).

The results of the multiple linear regression analysis and the backward stepwise elimination methods are presented in Tables 3 and 4, respectively.

The performance of a PPV, aphakia after the initial trauma, the loss of iris tissue, a penetrating injury, and the cutting of any prolapsed vitreous in the primary surgery are the independent variables with significant effects on the final visual outcome.

The comparisons of the initial BCVAs between the cases with and without these five significant independent variables are presented in Table 5.

The initial BCVAs were significantly reduced in cases with aphakia after the initial trauma, a loss of iris tissue, or an injury caused by blunt trauma.

The comparisons of the final BCVAs between the cases with or without these five significant independent variables are presented in Table 6.

The final BCVAs were significantly reduced in the patients who underwent PPV and in cases with aphakia after an initial trauma, the loss of iris tissue, or an injury caused by blunt trauma.

A regression equation obtained from the backward stepwise elimination was as follows:

Final BCVA = 0.914 + 0.502 × performance of PPV + 0.960 × aphakia after initial trauma + 0.831 × loss of iris tissue − 0.385 × penetrating injury − 0.506 × cutting any prolapsed vitreous in the primary surgery. The independent variables in the equation are Boolean indicator variables (1 or 0 for existence or nonexistence, resp.).

## 4. Discussion

In this study, a significant correlation was identified between the OTS and visual outcomes in one hundred thirty-one open globe injuries. Serious visual impairment was developed in approximately one-half of the cases with a BCVA below 0.1 Snellen. Independent variables other than the OTS parameters were analyzed. The eyes were evaluated in regard to our management (the number of surgeries, performance of PPVs, cutting of any prolapsed vitreous in the primary surgery, and time interval between the trauma and repair) and the findings at the initial surgery and during the follow-up visits. We demonstrated that the performance of PPVs, aphakia after the initial trauma, and the loss of iris tissue significantly inhibited the final visual outcomes, whereas the cutting of any prolapsed vitreous in the primary repair and penetrating-type injuries had positive effects on the final visual outcomes.

In this study, we reported that the age, number of surgeries performed, the eye tissue affected (corneal, scleral, or corneoscleral), the development of IVH, glaucoma or anterior segment inflammation, the presence of an intraocular foreign body, and the time interval between the trauma and repair did not have significant effects on the final BCVAs. Although the performance of PPVs decreased the final visual outcomes significantly, the total number of procedures did not significantly influence the final BCVAs. Procedures other than PPVs, specifically cataract surgeries and anterior segment revisions, could have improved the outcomes, which might have offset the poor prognosis in the patients who underwent a PPV. Although it is not always reliable, in many studies, the zone of injury is defined according to the location of the most posterior aspect of the injury [15]. Zone 1 injuries involve the cornea or the limbus, zone 2 injuries involve the anterior 5 mm of the sclera, and zone 3 injuries involve the more posterior aspects of the globe [15]. Zone 3 injuries are associated with a poor prognosis [11, 16]. Instead of using this classification, we simply noted whether the cornea, sclera, or corneasclera was affected. Detecting the injured part of the eye was straightforward and did not affect the final BCVAs. The performance of a PPV was one of the variables associated with a poor prognosis. Although a PPV was performed in thirteen of our cases for IVH and seven of our cases for an intraocular foreign body, the presence of these two indications was not a part of the regression equation we obtained. In several studies, IVH was correlated with a poor visual outcome [11, 17]. In a retrospective review of 180 patients at an emergency department in Portugal, IVH was associated with a poor outcome [18]. The medical management of glaucoma and anterior segment inflammation appears to prevent additional functional loss in our cases with high IOP and anterior segment inflammation because these two variables were eliminated with a backward stepwise elimination method. Therefore, these variables did not have a significant effect on our outcomes. The time between the trauma and the primary repair is an important prognostic factor, and visual outcomes were decreased for each day of delay [19, 20]. The majority of our cases were managed on a timely basis with a mean time interval of 13 hours between the trauma and the surgery, and we concluded that having the primary surgery within 13 hours would not influence the functional outcomes.

Retinal detachment is one of the parameters included in the OTS [6]. It was the most common indication for a PPV in our cases; therefore, it was reasonable to determine the performance of PPV as one of the prognostic factors according to the OTS [6]. However, there were other etiologies for the performance of PPVs, and we analyzed the performance of PPVs as an independent variable to evaluate its isolated effect. We demonstrated a significant increase in the possibility of having a worse visual prognosis in eyes that underwent a PPV, with a regression coefficient of 0.479. Proliferative vitreoretinopathy, ciliary body damage, and choroidal damage are common complications following ocular injuries and are associated with a poor prognosis [8, 21]. In addition to the surgical trauma, these findings could be associated with poor outcomes in cases in which PPV was performed. However, we did not investigate the existence of these findings in this study.

Aphakia after the initial trauma and the loss of iris tissue were associated with a poor final outcome. The presence of these two variables might reflect the severity of the injury. Lens extrusion and iris damage are associated with poor functional outcomes [7, 8, 10, 16, 19]. Aphakia after an initial trauma was noted in 20% of the eyes. Their initial and final BCVAs were worse than in those eyes without aphakia after an initial trauma. Lenticular involvement in children might lead to deprivational amblyopia [22, 23]. The amblyopia in our aphakic children could cause a further reduction in the visual outcomes. We considered traumatic cataracts managed secondarily as another variable. Some of the cases with traumatic cataracts underwent operation, whereas surgery was not recommended in other cases. Traumatic cataract was not included in our final regression equation. Consistent with our findings, in a report including one hundred thirty-one children, the need for cataract surgery was not found to be predictive of the visual outcome [24]. We might conclude that traumatic cataracts are not associated with a poor outcome when managed appropriately.

Globe rupture is one of the OTS parameters associated with poor visual outcomes [6]. A penetrating injury is defined as a single, full-thickness wound of the eye wall typically caused by a sharp object [15, 25]. The type of injury was penetrating in 32% of our cases. The BCVAs achieved in these cases were better than those in the blunt injury cases. The presence of a penetrating injury was one of the variables of our final regression equation. Blunt injuries causing globe ruptures are associated with a poor prognosis [6, 11]. In this study, the presence of a penetrating injury was one of the significant variables after the backward stepwise elimination. The cutting of any prolapsed vitreous in the primary surgery was correlated with a better functional outcome; however, the difference in the final BCVAs between the cases in which this procedure was applied and not applied did not reach statistical significance. The removal of any vitreous could reduce the risk of tractional retinal detachment and corneal decompensation [26, 27]. Prolapsed vitreous was identified and removed in seventeen cases. However, the severity of the ocular damage could have prevented visualization of prolapsed vitreous in some cases, which could be another factor associated with better outcomes in eyes that underwent prolapsed vitreous removal.

The strengths of this study include the long follow-up time, the evaluation of thirteen factors and their associations with visual outcomes using regression analyses, and the relatively large number of eyes included. There are some limitations associated with the evaluated factors. The aim of this study was to analyze variables other than the OTS parameters. Thus, we interpreted thirteen variables and obtained a regression equation that included the significant variables. The equation might be useful for estimating the final BCVAs; however, other possible predictors, such as the OTS parameters, hyphema, choroidal hemorrhage, and causes of the injuries, were not included in the model for the regression analyses. If these variables were evaluated, several of them could have been included in the equation. Another limitation associated with the possible predictors included was the consideration of these factors as independent variables for statistical purposes. The multicollinearity that was calculated supported the independence of the variables; however, some of these factors obviously affect each other, including the number of surgeries and the performance of PPVs. The majority of our cases were adult patients, and the age did not significantly affect our outcomes. If the percent of young cases was increased, age could have been one of the significant variables affecting outcomes. On the other hand, we could have analyzed children separately because of the unique problems in children's eyes after ocular trauma, mainly the risk of amblyopia [22].

## 5. Conclusions

According to the regression equation, the performance of PPV, aphakia after the initial trauma, and loss of iris tissue were associated with poor visual outcomes, whereas cutting any prolapsed vitreous in the primary repair and penetrating-type injury were associated with better visual outcomes. Further studies are required that should include additional possible predictors to obtain a more reliable equation for estimating the visual outcome.

## Figures and Tables

**Table 1:** The ocular trauma scores for one hundred thirty-one open glob injuries.

Ocular trauma score parameters	Number of cases (%)
Rupture of the globe	89 (68%)
Afferent pupillary defect	13 (10%)
Endophthalmitis	2 (2%)
Retinal detachment	24 (18%)
Perforating injury	5 (4%)
Initial vision ≥ 5/10 Snellen	1 (0.7%)
Initial vision 4/10–1/10 Snellen	10 (7%)
Initial vision 19/200–1/100 Snellen	13 (10%)
Hand movement and light perception	100 (77%)
No light perception	7 (5%)

**Table 2:** Thirteen independent variables evaluated among one-hundred thirty-one open globe injuries.

Independent variables	Findings (numerical/categorical)
Age	35.24 ± 21.6 (4–88) years
Number of surgeries	
1	67 (51%)
2	47 (36%)
3	17 (13%)
Performance of pars plana vitrectomy	32 (24%)
Lens status	
No pathology	50 (38%)
Traumatic cataract	55 (42%)
Aphakia after initial trauma	26 (20%)
Affected tissue	
Corneal	59 (45%)
Scleral	17 (13%)
Corneoscleral	55 (42%)
Intravitreal hemorrhage	29 (22%)
Intraocular foreign body	17 (13%)
Glaucoma during follow-up	9 (7%)
Anterior segment inflammation during follow-up	23 (18%)
Loss of iris tissue	61 (47%)
Penetrating injury	42 (32%)
Cutting any prolapsed vitreous in the primary surgery	17 (13%)
Time interval between the trauma and repair	13.5 ± 13.2 (6–96) hours

**Table 3:** The influences of thirteen independent variables on the final visual outcome using the multiple linear regression analysis.

Independent variable	Regression coefficient	*P* value
Constant	0.740	0.017^*∗*^
Number of surgeries	0.049	0.793
Age	0.008	0.051
Performance of pars plana vitrectomy	0.440	0.106
Lens status^1^		
Traumatic cataract (L1)	0.193	0.401
Aphakia after initial trauma (L2)	1.038	<0.001^*∗*^
Affected tissue^2^		
Scleral (E1)	−0.052	0.842
Corneoscleral (E2)	0.000	0.998
Intravitreal hemorrhage	−0.251	0.189
Intraocular foreign body	−0.086	0.707
High intraocular pressure during follow-up	−0.486	0.108
Anterior segment inflammation during follow-up	−0.048	0.805
Loss of iris tissue	0.769	<0.001^*∗*^
Penetrating injury	−0.376	0.027^*∗*^
Cutting any prolapsed vitreous in the primary surgery	−0.607	0.012^*∗*^
Time interval between the trauma and repair	−0.008	0.166

^*∗*^*P* < 0.05. ^1^If L1 = 0 and L2 = 0, the independent variable was no lenticular pathology. If L1 = 1 and L2=0, the independent variable was traumatic cataract. If L1 = 0 and L2 = 1, the independent variable was aphakia during primary surgery.

^2^If E1 = 0 and E2 = 0, the independent variable was corneal perforation. If E1 = 1 and E2 = 0, the independent variable was scleral perforation. If E1 = 0 and E2 = 1, the independent variable was corneoscleral perforation.

**Table 4:** The significant independent variables after the backward stepwise elimination.

Independent variable	Regression coefficient	*P* value
Constant	0.914	<0.001^*∗*^
Performance of pars plana vitrectomy	0.502	0.002^*∗*^
Aphakia after initial trauma (L2)^1^	0.960	<0.001^*∗*^
Loss of iris tissue	0.831	<0.001^*∗*^
Penetrating injury	−0.385	0.014^*∗*^
Cutting any prolapsed vitreous in the primary surgery	−0.506	0.021^*∗*^

^*∗*^*P* < 0.05. ^1^If L1 (the presence of traumatic cataract) = 0 and L2 = 1, the independent variable was aphakia during primary surgery.

**Table 5:** The comparisons of the initial visual acuities among cases with and without the significant independent variables after the backward stepwise elimination.

Variable	Number of cases	Initial VA in logMAR	*P* value
Performance of pars plana vitrectomy			
+	32	2.50 ± 0.60	0.771
−	99	2.46 ± 0.61	
Aphakia after initial trauma			
+	26	2.76 ± 0.07	<0.001^*∗*^
−	105	2.40 ± 0.66	
Loss of iris tissue			
+	61	2.63 ± 0.45	0.004^*∗*^
−	70	2.33 ± 0.69	
Penetrating injury			
+	42	2.29 ± 0.78	0.044^*∗*^
−	89	2.56 ± 0.49	
Cutting any prolapsed vitreous in the primary surgery			
+	17	2.52 ± 0.41	0.730
−	114	2.46 ± 0.63	

^*∗*^*P* < 0.05.

**Table 6:** The comparisons of the final visual outcomes among cases with and without the significant independent variables after the backward stepwise elimination.

Variable	Number of cases	Initial VA in logMAR	*P* value
Performance of pars plana vitrectomy			
+	32	1.92 ± 0.93	0.003^*∗*^
−	99	1.26 ± 1.10	
Aphakia after initial trauma			
+	26	2.54 ± 0.57	<0.001^*∗*^
−	105	1.15 ± 1.02	
Loss of iris tissue			
+	61	2.02 ± 0.93	<0.001^*∗*^
−	70	0.92 ± 0.97	
Penetrating injury			
+	42	0.98 ± 0.97	0.001^*∗*^
−	89	1.64 ± 1.10	
Cutting any prolapsed vitreous in the primary surgery			
+	17	1.08 ± 0.89	0.105
−	114	1.48 ± 1.12	

^*∗*^*P* < 0.05.
